# Podocyte Injury and Albuminuria in Experimental Hyperuricemic Model Rats

**DOI:** 10.1155/2017/3759153

**Published:** 2017-02-28

**Authors:** Shinichiro Asakawa, Shigeru Shibata, Chikayuki Morimoto, Takeshi Shiraishi, Takashi Nakamura, Yoshifuru Tamura, Takanori Kumagai, Makoto Hosoyamada, Shunya Uchida

**Affiliations:** ^1^Division of Nephrology, Department of Internal Medicine, Teikyo University School of Medicine, 2-11-1 Kaga, Itabashi-ku, Tokyo 173-8605, Japan; ^2^Support for Community Medicine Endowed Chair, Teikyo University School of Medicine, Tokyo 173-8605, Japan; ^3^Pharmacological Study Group, Pharmaceutical Research Laboratories, Sanwa Kagaku Kenkyusho, Mie 511-0406, Japan; ^4^Department of Human Physiology and Pathology, Faculty of Pharma-Sciences, Teikyo University, 2-11-1 Kaga, Itabashi-ku, Tokyo 173-8605, Japan

## Abstract

Although hyperuricemia is shown to accelerate chronic kidney disease, the mechanisms remain unclear. Accumulating studies also indicate that uric acid has both pro- and antioxidant properties. We postulated that hyperuricemia impairs the function of glomerular podocytes, resulting in albuminuria. Hyperuricemic model was induced by oral administration of 2% oxonic acid, a uricase inhibitor. Oxonic acid caused a twofold increase in serum uric acid levels at 8 weeks when compared to control animals. Hyperuricemia in this model was associated with the increase in blood pressure and the wall-thickening of afferent arterioles as well as arcuate arteries. Notably, hyperuricemic rats showed significant albuminuria, and the podocyte injury marker, desmin, was upregulated in the glomeruli. Conversely, podocin, the key component of podocyte slit diaphragm, was downregulated. Structural analysis using transmission electron microscopy confirmed podocyte injury in this model. We found that urinary 8-hydroxy-2′-deoxyguanosine levels were significantly increased and correlated with albuminuria and podocytopathy. Interestingly, although the superoxide dismutase mimetic, tempol, ameliorated the vascular changes and the hypertension, it failed to reduce albuminuria, suggesting that vascular remodeling and podocyte injury in this model are mediated through different mechanisms. In conclusion, vasculopathy and podocytopathy may distinctly contribute to the kidney injury in a hyperuricemic state.

## 1. Introduction

Chronic kidney disease (CKD) continues to be a public health problem worldwide [1]. CKD not only causes end-stage renal disease (ESRD) but also increases the prevalence of cardiovascular disease [2, 3] and, therefore, early intervention against the risk factors for CKD is crucial to improve renal and cardiovascular outcomes. Hyperuricemia has long been speculated as a possible risk factor of the incidence and progression of CKD over the last decade, but without reaching a broad consensus [4–7]. The reasons of inconsistent results are ascribed to the differences in the enrolled participants, observation periods, endpoints studied, and particularly the presence or absence of confounders. Moreover, the time-varying nature or trajectory of serum uric acid (UA) has been completely neglected in the previous study methods, and the risk of serum UA may be too subtle to be independently detected in the existence of highly influential risk factors such as proteinuria and hypertension [8]. We recently showed that the effect of serum UA in the follow-up influenced the incipient ESRD by a propensity score analysis and that serum UA should be kept less than 6.5 mg/dL to inhibit the renal outcome [8].

Several interventional randomized controlled trials (RCT) revealed the significant inhibition of decline in estimated glomerular filtration rate (eGFR) by allopurinol, a xanthine oxidase (XO) inhibitor, but the small number of participants and short observation duration hampered the definite conclusion [9–11]. Only one recent study successfully showed that allopurinol inhibited reaching renal endpoints of doubling of serum creatinine and incidence of ESRD by the time-to-event analysis [12]. A double-blind RCT recruiting more than 400 participants is under way in Japan using a recently developed novel XO inhibitor, febuxostat [13].

Together with clinical evidence, experimental studies providing mechanistic insights of UA-caused kidney injuries are necessary. A rat model receiving oxonic acid, an inhibitor of uricase, has been widely used to study the pathophysiological roles of hyperuricemia [14–17]. These studies provided insights into the mechanisms for cardiovascular injury associated with hyperuricemia and demonstrated that UA directly causes vascular injury and hypertension via crystal-independent mechanisms [15, 16, 18]. Importantly, although UA is a strong antioxidant in the plasma [19], hyperuricemia accelerates target organ damage through the prooxidant property of UA [20]. In vascular endothelial cells, oxidative stress associated with high UA levels decreased endothelial nitric oxide, leading to endothelial dysfunction [21]. Recent studies also demonstrated the role of oxidative stress in systemic hypertension associated with hyperuricemia [17, 21]. Thus far, however, whether hyperuricemia causes kidney damage solely via vascular injury remains unclear. Of note, previous studies demonstrated that hyperuricemia aggravates proteinuria in the rat remnant kidney model [22], although the mechanisms remain largely obscure.

Glomerular visceral epithelial cells, or podocytes, are present outside the glomerular basement membrane and serve as the filtration barrier to prevent the leak of plasma proteins into the urine. These cells constitute characteristic interdigitating foot processes, which are connected to each other by the slit diaphragm proteins such as podocin and other molecules [23–25]. Because the normal formation of podocyte slit diaphragms is the integral part of the glomerular permselectivity, its dysregulation constitutes a major cause of pathological proteinuria [26]. Interestingly, accumulating data revealed that podocytopathy plays a fundamental role in kidney diseases associated with metabolic disorders such as diabetic kidney disease, salt-sensitive hypertension, and obesity-related glomerulopathy [27–31]. However, despite the possible link between hyperuricemia and CKD, little is known on the role of hyperuricemia in modulating podocyte function. Thus, we set out to examine the increase in albuminuria and the involvement of podocytes in the kidney injury caused by experimental hyperuricemia in conjunction with the involvement of oxidative stress.

## 2. Materials and Methods

### 2.1. Animal Experiments

All animal experiments were performed in accordance with the Institute Animal Care and Use Committee of the Teikyo University (Teikyo University School of Medicine Animal Ethics Committee #14-035). Male Sprague-Dawley rats weighing 200 g were purchased from Sankyo Lab (Tokyo, Japan). Rats were divided into two groups after body weight, urine collection and blood pressure measurement. One group received standard diet (CRF1, Oriental Yeast, Tokyo, Japan) (*n* = 13), whereas the other group received oxonic acid (Sigma, St. Louis, MO, USA) mixed in the diet (2 g/100 g chow; the dose was decided according to previous studies) (*n* = 12) [18]. Body weight and blood pressure were measured at 4 and 8 weeks. Urine was collected for 24 hours using individual metabolic cages at 4 and 8 weeks. At 8 weeks, animals were euthanized under anesthesia using inhaled isoflurane.

In another set of experiments, rats received oxonic acid (2 g/100 g chow) and tempol, a superoxide dismutase mimetic (*n* = 8). Tempol was administered via drinking water at a concentration of 1 mmol/L, which is shown to be effective in several rodent models [32, 33]. Rats that received oxonic acid and normal water (*n* = 7) were used as controls to evaluate the protective effects of tempol. After blood pressure measurement and urine collection, animals were euthanized at 8 weeks.

Systolic blood pressure of conscious rats was measured by the tail-cuff method. Blood samples were obtained by cardiac puncture. Kidneys were removed, snap-frozen, and stored at −80°C until use. Urinary albumin levels were measured by ELISA (SRL, Tokyo, Japan). SDS-PAGE analysis of the urine was performed as described previously [34].

Serum UA concentrations were determined using high-performance liquid chromatograph equipped with a UV spectrophotometric detector (Prominence; Shimadzu, Kyoto, Japan). UA standard was dissolved in water by adding 2 mmol/L ammonium hydroxide solution (final concentration of 5 mg/dL). Serum samples were centrifuged and filtered through a Millipore filter (0.22 *μ*m pore size; Darmstadt, Germany). Samples were injected onto a Wakosil GP-N6 column (15 × 4.6 mm ID) with mobile phase of 98% (v/v) 0.2 mol/L sodium phosphate buffer, pH6.0, and 2% (v/v) acetonitrile at a flow rate of 0.5 mL/min. Under these conditions typical retention time for uric acid (detected at 284 nm) was 3.68 min.

### 2.2. Immunohistochemistry and Quantification

Kidney tissues were fixed in 4% paraformaldehyde in PBS at 4°C. Tissues were incubated in 30% sucrose in PBS overnight at 4°C and mounted in OCT (Tissue-Tek, Tokyo, Japan) for sectioning [34]. After blocking, tissue sections were stained with the indicated primary antibodies and affinity-purified secondary antibodies-conjugated HRP (DAKO, Glostrup, Denmark). Primary antibodies used included antibodies against *α*SMA (Sigma), desmin (DAKO, Glostrup, Denmark), podocin (Abcam, Cambridge, MA, USA), and 8-hydroxy-2′-deoxyguanosine (8OHdG) (JaICA, Shizuoka, Japan). Quantitative analysis of afferent arterioles was performed as previously described [16]. For afferent arterioles, vessels with internal elastic lamina adjacent to glomeruli were selected. Arcuate arteries were identified by the location at the border of renal cortex and medulla. Areas positive for *α*SMA in the cross section of these vessels were quantitated using NanoZoomer (Hamamatsu Photonics, Hamamatsu, Japan) and Aperio ImageScope (Leica, Buffalo Grove, IL, USA). For quantification of desmin, podocin, and 8OHdG in the glomeruli, the percentage of positive area was determined as positive pixels per total pixels in a glomerulus using Image Scope software. For each rat, 20 glomeruli were randomly analyzed.

### 2.3. Transmission Electron Microscopy

Ultramicrostructure of the glomeruli was observed by transmission electron microscopy. Small pieces of cortex were fixed in 2.5% glutaraldehyde, dehydrated through graded ethanol and propylene oxide, and embedded in Epon 812 using standard procedures. Ultrathin sections were stained with uranyl acetate and with Reynolds lead citrate. The specimens were observed using Hitachi transmission electron microscope H-7650 (Hitachi Science Systems Ltd., Hitachinaka, Japan).

### 2.4. XO Activity Measurement in the Kidney Cortex

XO activity was measured as xanthine oxidoreductase (XOR) activity using a method described previously [35]. In brief, the kidney cortex was homogenized in phosphate buffered saline (pH 7.4) containing protease inhibitor cocktail (Roche, Basel, Switzerland) and centrifuged at 20,000 ×g, 4°C for 20 min. The kidney homogenates were added to mixture containing [^15^N_2_] xanthine (0.4 mmol/L), NAD^+^ (0.4 mmol/L), and oxonate (0.013 mmol/L) in 20 mmol/L Tris buffer (pH 8.5) and were incubated at 37°C for 30 min. Subsequently, the mixtures were mixed with 500 *μ*L of methanol containing [^13^C_2_, ^15^N_2_] uric acid as internal standard and centrifuged at 20,000 ×g for 10 min at 4°C. The supernatants were transferred to new tubes and dried using centrifugal evaporator. The residues were reconstituted with 150 *μ*L of distilled water, filtered through an ultrafiltration membrane (Amicon Ultra 0.5 centrifugal filter devices, 3K, Millipore, Merck KGaA, Darmstadt, Germany), and the [^15^N_2_] uric acid production was measured with LC/MS (LTQ-Orbitrap, Thermo Fisher Scientific, Waltham, MA, USA). Each activity was expressed as [^15^N_2_] uric acid production nmoL/min/mg protein.

### 2.5. Statistical Analysis

All data are continuous variables and thus expressed as mean ± standard deviation (SD). Based on the distribution of the data, the parametric statistics were utilized. Unpaired *t*-test was used for comparisons between two groups. Correlation of between parameters was analyzed by Pearson's correlation test. A value of *P* < 0.05 was considered statistically significant.

## 3. Results and Discussion

### 3.1. Blood Pressure Elevation and Renal Vasculopathy in Experimental Hyperuricemia

**Figure 1:**
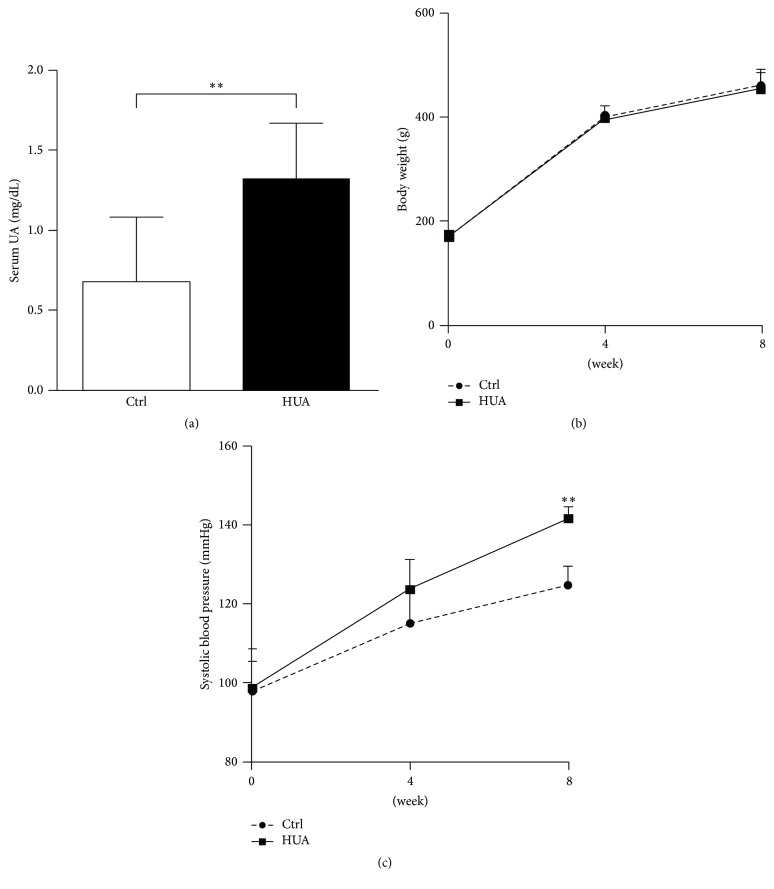
Blood pressure elevation in oxonic acid-treated hyperuricemic rats. (a) Serum uric acid (UA) levels were measured by high-performance liquid chromatography at 8 weeks in control rats (Ctrl) and hyperuricemic rats (HUA) receiving 2% oxonic acid. (b) Body weight in control (Ctrl) and hyperuricemic (HUA) rats. Body weight did not differ throughout the experiment. (c) Systolic blood pressure was significantly higher in HUA group than Ctrl at 8 weeks. Data are expressed as mean ± SD; *n* = 12 or 13 per group. ^*∗∗*^*P* < 0.01.

To evaluate the mechanism whereby hyperuricemia impairs kidney function, we orally administered oxonic acid (OA), the uricase inhibitor, to male Sprague-Dawley rats [18]. This model causes hyperuricemia without increasing purine metabolism and, therefore, in theory, without increasing XO activity. As shown in Table 1 and Figure 1(a), OA successfully increased serum UA levels at 8 weeks compared with the control group (*P* = 0.002). Body weight was similar between the two groups (Figure 1(b)), indicating that OA did not affect food and water intake. Consistent with the previous report [18], hyperuricemia caused a moderate increase in systolic blood pressure when compared to the control group at 8 weeks (*P* < 0.001) (Table 1 and Figure 1(c)).

**Figure 2:**
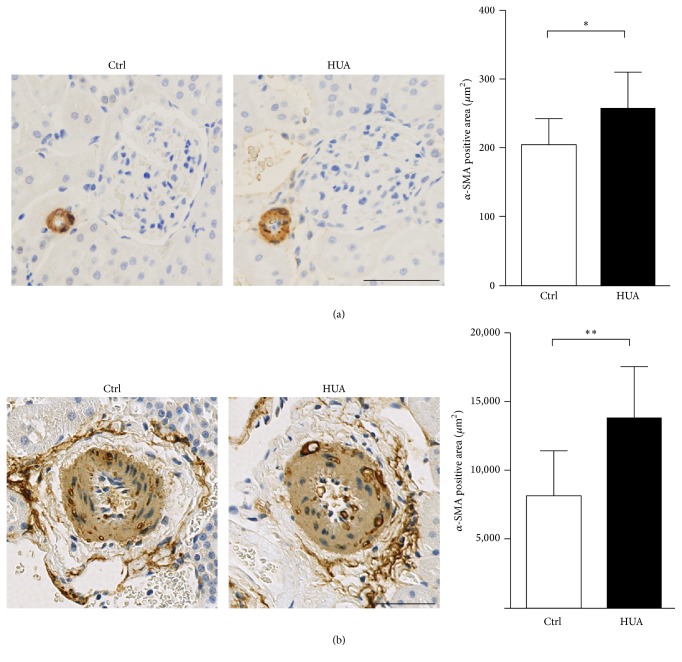
Thickening of afferent arterioles and arcuate arteries in hyperuricemic rats. (a and b) Rat kidney sections were stained for *α*-smooth muscle actin (*α*-SMA) to evaluate the thickening of afferent arterioles (a) and arcuate arteries (b). Bars represent 50 *μ*m. Bar graphs show the results of quantitation. Data are expressed as mean ± SD; *n* = 6 for each group. ^*∗*^*P* < 0.05; ^*∗∗*^*P* < 0.01.

Clinical and experimental studies indicated that hyperuricemia is associated with renal arteriolopathy [15, 18, 36]. We next examined whether hyperuricemia induces arteriopathy, as well as arteriolopathy. Kidney sections from control and hyperuricemic rats were stained with *α*-smooth muscle actin (*α*SMA), a marker for vascular smooth muscle cells (VSMC) to evaluate vascular hypertrophy at the levels of afferent arterioles and arcuate arteries. As shown in Figure 2(a), hyperuricemia caused thickening of afferent arterioles in hyperuricemic rats compared to control rats (*P* = 0.043). The vascular changes were not limited to the arterioles but were also present in the medium-sized arteries, as demonstrated by the increased *α*SMA staining in renal arcuate arteries (*P* = 0.004; Figure 2(b)). In addition to the confirmation of the finding of the arterioles [16, 36], we could demonstrate the involvement of medium-sized arteries in the vasculopathy induced by hyperuricemia.

### 3.2. Podocyte Injury Is Involved in Hyperuricemic Rats

**Figure 3:**
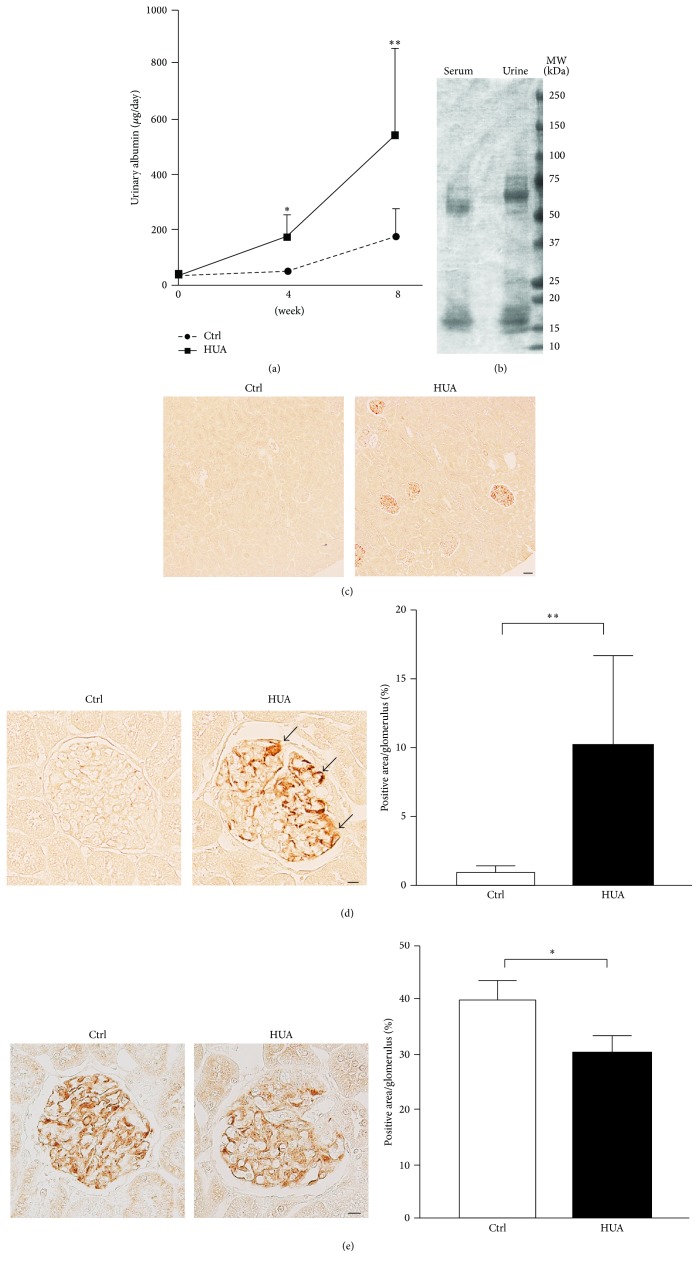
Involvement of podocyte injury in hyperuricemic rats. (a) Urinary albumin excretion measured at 8 weeks in control (Ctrl) and hyperuricemic (HUA) rats. (b) SDS-PAGE analysis of serum and urine from hyperuricemic rats. (c) and (d) Representative micrographs of immunostaining for desmin, a marker for podocyte injury, at low (c) and high (d) magnifications. Bar graphs show the quantitative evaluation of desmin staining in the glomeruli. (e) Representative micrographs of immunostaining for podocin, a component of the podocyte slit diaphragm. Bar graphs show the quantitative evaluation of podocin staining in the glomeruli. Data are expressed as mean ± SD; *n* = 12 or 13 for (a); *n* = 6 or 7 for (d); and *n* = 3 for (e). Bars represent 50 *μ*m (c) and 10 *μ*m (d, e). ^*∗*^*P* < 0.05; ^*∗∗*^*P* < 0.01. Arrows indicate increased desmin staining in podocytes.

Given the experimental evidence that hyperuricemia facilitates the progression of kidney injury in the rat remnant kidney model [22], we next determined whether hyperuricemia per se (i.e., without nephrectomy) causes kidney damage. Although the serum creatinine levels did not significantly differ between control and hyperuricemic rats (Table 1; *P* = 0.804), albuminuria progressively increased in hyperuricemic rats (Figure 3(a) and Table 1); the urinary albumin levels were significantly higher at as early as 4 weeks (*P* = 0.049) and further increased at 8 weeks (*P* = 0.0015). SDS-PAGE analysis of urine obtained from hyperuricemic rats resembled the electrophoresis pattern of serum proteins, consistent with nonselective glomerular proteinuria (Figure 3(b)).

**Figure 4:**
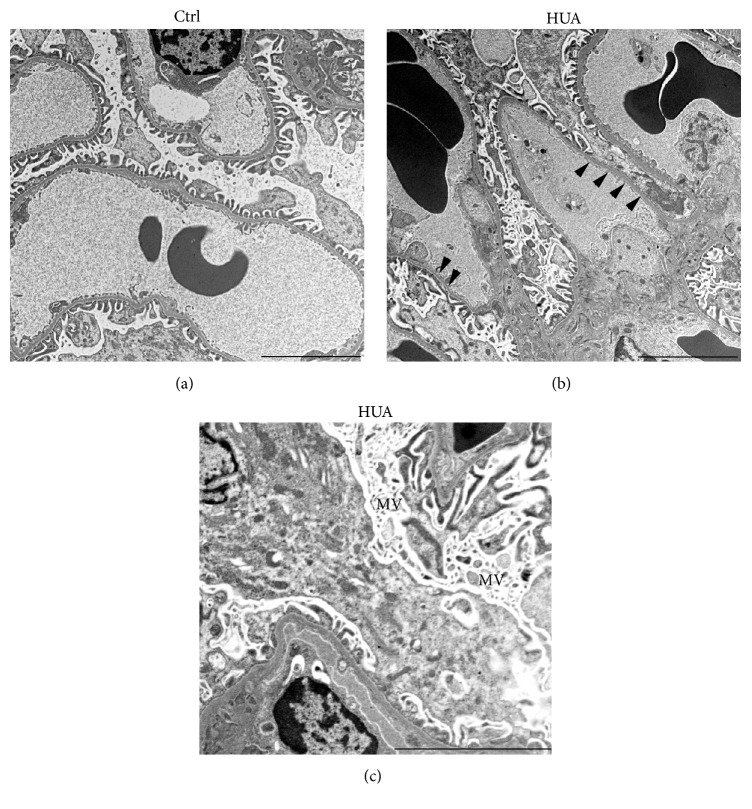
Podocyte injury in hyperuricemic rats is confirmed by electron microscopy. Transmission electron micrographs of podocyte foot process in the glomeruli of indicated animals. Podocytes in the kidney from hyperuricemic rats (HUA) showed foot process effacement (arrowheads) and microvillus transformation (MV). These changes were less evident in the control (Ctrl) group. Bars represent 5 *μ*m.

By forming foot processes and slit diaphragms, podocytes play central roles to prevent albuminuria in a normal state. To determine the cause of increased urinary albumin in this model, we analyzed the involvement of podocytes. Interestingly, desmin, a sensitive podocyte injury [27, 37], was upregulated in podocytes of hyperuricemic rats, but not in those of control rats (Figures 3(c) and 3(d)). Moreover, immunostaining of the slit diaphragm component podocin revealed that it was significantly decreased in hyperuricemic rats (Figure 3(e)). Consistent with these findings, a structural analysis using transmission electron microscopy demonstrated the occasional retraction of the podocyte foot processes in hyperuricemic rats (Figure 4). Podocytes from hyperuricemic rats also showed a sign of microvillus transformation (Figure 4(c)), indicating podocyte damage [38]. These data are consistent with the immunohistochemical analysis and demonstrate that podocytopathy underlies the increase in albuminuria in the hyperuricemic model.

### 3.3. Role of Oxidative Stress in Vasculopathy and Podocyte Injury Induced by Hyperuricemia

**Figure 5:**
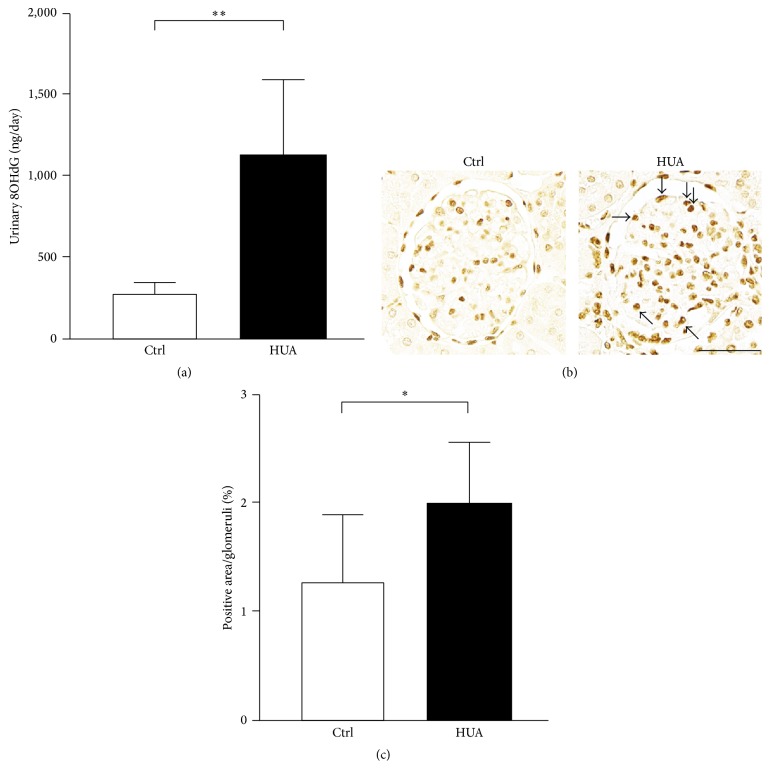
Involvement of oxidative stress in the glomerular injury in hyperuricemic rats. (a) Urinary levels of 8-hydroxy-2′-deoxyguanosine (8OHdG) were measured in control (Ctrl) and hyperuricemic rats (HUA). (b) Staining of 8OHdG in the glomeruli. Arrows indicate the enhancement of the staining. Bar represents 50 *μ*m. (c) Quantitative evaluation of 8OHdG staining in the glomeruli. 8OHdG-positive nuclei were counted as percentage of total glomerular nuclei. Data are expressed as mean ± SD; *n* = 12 for (a); *n* = 7 for (c). ^*∗*^*P* < 0.05; ^*∗∗*^*P* < 0.01.

The above data indicate that podocyte is involved in the kidney injury associated with hyperuricemia, resulting in increased urinary albumin excretion. Multiple lines of evidence demonstrate the importance of oxidative stress in podocyte dysfunction, especially when associated with endocrine and metabolic disorders [27, 31, 39]. To elucidate the possible mechanisms underlying podocyte injury in hyperuricemic rats, we examined the expression of oxidative stress markers in our model. Remarkably, urinary 8OHdG levels were as much as 4-fold higher in hyperuricemic rats compared with control rats (1,130 ± 466 ng/day in hyperuricemic group versus 281 ± 80 ng/day in control group; *P* < 0.001) (Figure 5(a)). Moreover, the immunostaining of the kidney cortex revealed that 8OHdG staining was upregulated in glomerular cells, including podocytes (Figures 5(b) and 5(c)).

**Figure 6:**
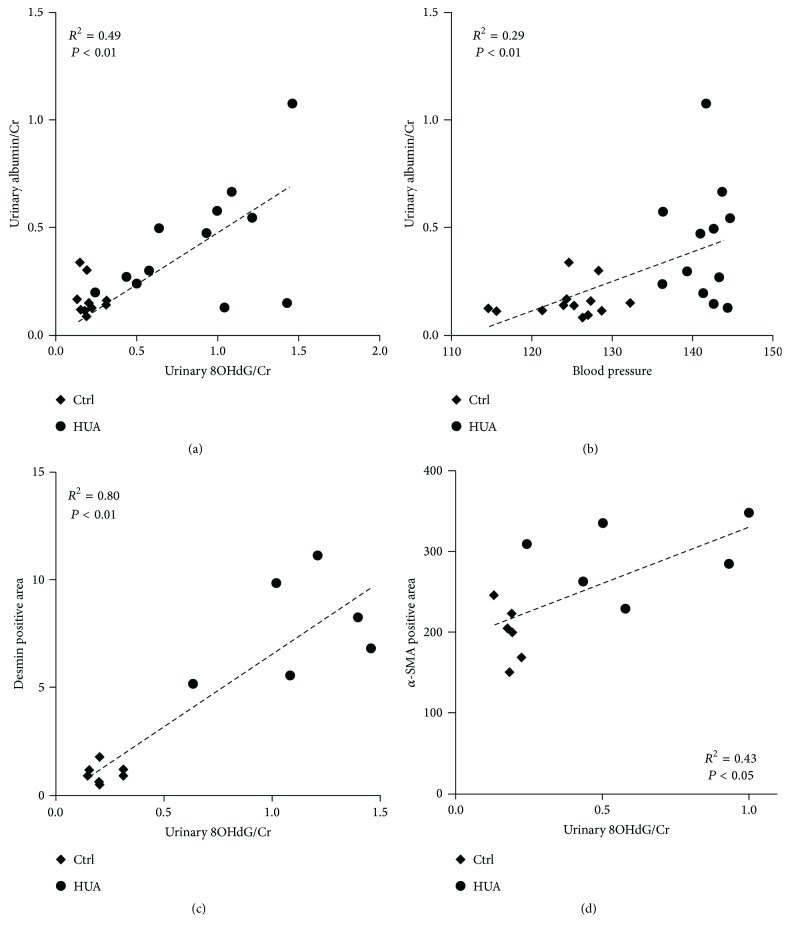
Correlation of different parameters in control and hyperuricemic rats. (a and b) Urinary albumin levels correlated with urinary 8OHdG levels (a) and systolic blood pressure (b). The coefficient of determination was 0.49 for (a) and 0.29 for (b). (c and d) Correlation between urinary 8OHdG levels and the degree of podocytopathy, as determined by desmin-positive area (c), or the degree of arteriolopathy, as determined by the afferent arteriole thickness (d).

Correlation analysis revealed that albuminuria correlated with urinary 8OHdG levels (*R*^2^ = 0.49; Figure 6(a)). Albuminuria was also positively correlated with blood pressure levels, although to a lesser extent (*R*^2^ = 0.29; Figure 6(b)). We further evaluated the relationship between urinary 8OHdG and the indices of podocyte injury and vascular remodeling. Of note, the index of podocytopathy was highly correlated with urinary 8OHdG levels (*R*^2^ = 0.80; Figure 6(c)), further supporting the involvement of reactive oxygen species in podocyte damage. Urinary 8OHdG levels also correlated with the degree of arteriolopathy but to a lesser extent (*R*^2^ = 0.43; Figure 6(d)).

**Figure 7:**
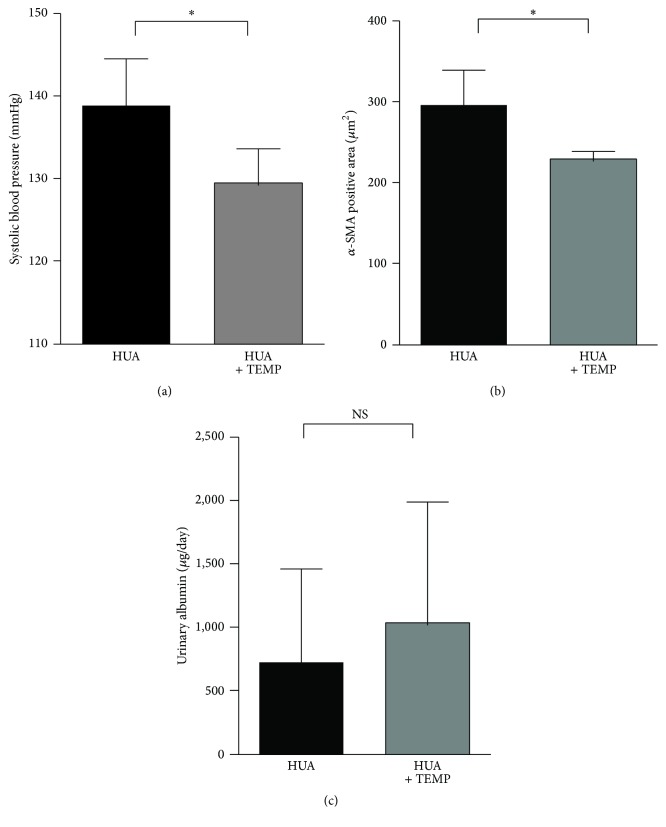
Superoxide dismutase (SOD) mimic tempol ameliorated hypertension but did not reduce albuminuria in hyperuricemic rats. (a–c) Systolic blood pressure (a), thickening of afferent arterioles as assessed by *α*-smooth muscle actin staining (b), and urinary albumin (c) in HUA rats with and without coadministration of tempol. Data are expressed as mean ± SD; *n* = 7 for HUA and 8 for HUA + TEMP (a and c); *n* = 4 for (b). ^*∗*^*P* < 0.05; NS: not significant.

Previous studies reported that tempol, a superoxide dismutase mimetic, successfully ameliorated vascular damage and blood pressure elevation in experimental hyperuricemia [17, 40]. To test whether podocyte injury in our model was induced by similar mechanisms, we administered tempol to hyperuricemic rats. Consistent with previous reports [17, 40], systolic blood pressure levels were significantly lower in the hyperuricemia plus tempol group than in the hyperuricemia group (Figure 7(a)). Quantitative analysis using *α*SMA staining demonstrated that tempol also ameliorated the thickening of the afferent arterioles (Figure 7(b)). Interestingly, however, urinary albumin levels were not altered by tempol (Figure 7(c)), despite the reduced blood pressure and the prevention of vasculopathy. This discrepancy indicates that hyperuricemia-induced podocyte injury and the resultant albuminuria may occur independently of vascular dysfunction and hypertension.

**Figure 8:**
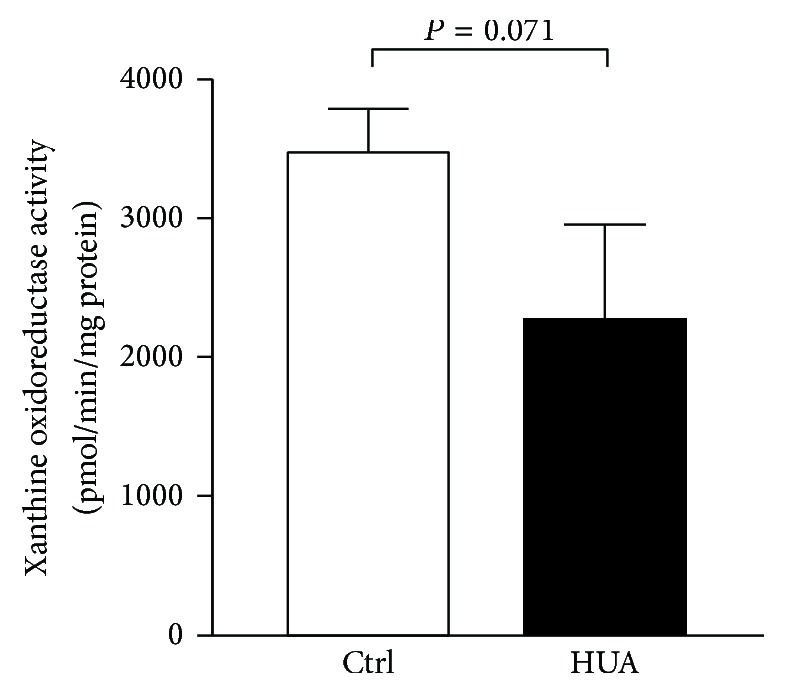
Xanthine oxidoreductase activity in the kidney. Levels of xanthine oxidoreductase activity were measured in the kidneys from control (Ctrl) and hyperuricemic rats (HUA).

To investigate the role of XO activity in the present model, we compared XOR activity in the kidney of control and hyperuricemic rats. As shown in Figure 8, XOR activity was not elevated but rather tended to be decreased in hyperuricemic rats compared with control rats.

### 3.4. Discussion

In this study, we demonstrated that the experimental hyperuricemia induced by uricase inhibition is associated with podocyte injury and significant albuminuria. Podocyte injuries were confirmed by the increase in desmin expression in podocytes and by slit membrane abnormalities, including decreased podocin expression and augmented foot process effacement assessed by transmission electron microscopy. Podocyte injury can be related to activation of cellular oxidative stress, given that the degrees of albuminuria and desmin staining correlated with urinary 8OHdG excretion. Of interest, tempol ameliorated high blood pressure and vascular remodeling, corroborating with the previous reports [17, 40], but failed to reduce albuminuria. Although the previous studies did not investigate the relation between albuminuria and tempol, the present results may indicate that arteriolopathy and podocytopathy occur via distinct mechanisms in hyperuricemic state. A possible pathological mechanism may be attributed to mitochondrial alterations and decreased intracellular ATP concentrations [21], but more studies are needed to explore the hyperuricemia-induced podocyte injury independently of superoxide-mediated mechanism.

The involvement of oxidative stress in hyperuricemia-induced kidney injury was recently emphasized in the context of XO inhibition with an advent of novel XO inhibitors such as febuxostat and topiroxostat [20]. The activity of XO generates superoxide in the cells, leading to cell damage, which can be abrogated by treatment with XO inhibitors [35, 41]. In fact, topiroxostat significantly reduced urinary albumin excretion by 33% of the baseline at 22 weeks in patients with stage 3 CKD [13]. Similarly, febuxostat treatment of patients with stage 3 CKD could significantly decrease the albuminuria and proteinuria in 12 weeks [42]. In theory, however, it was supposed that our hyperuricemic rat model induced by uricase inhibitor may not necessarily be associated with the increase in XO activity. Indeed, XOR activity measured in the kidney was not elevated or rather tended to decrease probably due to the product inhibition in hyperuricemic model rats [43]. Therefore, UA itself but not XO activity may play a critical role in the kidney injuries seen in the present study. Nonetheless, given that kidneys are composed of many different cell types, our data do not exclude the possibility that XO activity is increased in specific cell types including podocytes. It is generally accepted that extracellular (circulating) UA is the most abundant aqueous antioxidant in humans and serves as the major free radical scavenger in plasma [19]. The extracellular UA is particularly effective in quenching hydroxyl, superoxide, and peroxynitrite radicals and may serve a protective physiological role by preventing lipid peroxidation [19]. On the other hand, UA loses its radical scavenging activity under hydrophobic conditions [44] and can become a strong prooxidant within the cells. Indeed, recent studies indicate that UA once enters into intracellular compartment increases reactive oxygen species [21, 45, 46]. Consistent with these data, we found that hyperuricemia caused glomerular injury, which was associated with increased 8OHdG levels. Taken all together, UA may serve either a scavenger or a prooxidant depending on the biological milieu. In addition, two distinct pathophysiological scenarios by UA itself and by XO-mediated chain of events may underlie the pathophysiology of cellular damage associated with hyperuricemia.

Another question arises how intracellular UA is increased in response to the increase in serum UA. One possibility is that UA is taken up by a sort of urate transporter(s). Since the discovery of the first urate transporter, URAT1, in the renal proximal tubular cells [47], accumulating evidence indicates that URAT1 is also present in other cell types such as vascular smooth muscle cells (VSMC) [48] and endothelial cells [49]. In VSMC, UA enters the cells via URAT1, resulting in the activation of transcription factors and cytokines, including nuclear factor-*κ*B, activator protein-1, and monocyte chemoattractant protein-1, ultimately leading to VSMC proliferation and vascular dysfunction [22, 48]. More recent data indicate that UA can induce signaling in renal mesangial cells [50] and collecting duct cells [14]. Thus, it is possible that UA enters into glomerular podocytes, leading to tissue damage and resultant albuminuria in the setting of hyperuricemia. Anyhow, future studies are warranted to explore the mechanisms whereby intracellular UA modulates podocyte function.

A potential limitation of our study is that we did not provide detailed mechanisms of glomerular podocyte injury observed in our model. Future studies using cell culture are necessary to evaluate the causal role of UA and downstream signaling in podocyte damage. Biological actions of oxonic acid other than uricase inhibition may also be considered, although it is widely used to study the effects of hyperuricemia in rats.

## 4. Conclusion

The present study demonstrates that hyperuricemia in rats induces albuminuria associated with podocyte injury. Our data indicate that hyperuricemia can play a causal role in the progression of CKD not only by promoting circulatory system abnormalities, but also by increasing albuminuria, one of the most influential risk factors. Therefore, these data indicate the importance of appropriately controlling serum UA to prevent decline in kidney function in patients with CKD.

## Figures and Tables

**Table 1:** Biological parameters in control and hyperuricemic (HUA) rats at 8 weeks.

	Control	HUA
Body weight, g	459.2 ± 25.5	453.3 ± 38.5
Blood pressure, mmHg	124.6 ± 5.0	141.6 ± 2.7^*∗∗*^
Urine volume, mL	13.3 ± 7.0	14.4 ± 6.3
Urinary albumin, *μ*g/day	177.0 ± 102.4	543.7 ± 317.1^*∗∗*^
Serum UA, mg/dL	0.67 ± 0.42	1.32 ± 0.35^*∗∗*^
Serum Cr, mg/dL	0.34 ± 0.02	0.33 ± 0.02

Values are means ± SD, ^*∗∗*^*P* < 0.01 versus control.
